# Civilian vascular trauma, treatment and outcome at a level 1-trauma centre

**DOI:** 10.1186/s13049-022-01059-5

**Published:** 2022-12-21

**Authors:** B. K. Johannesdottir, T. Geisner, E. T. Gubberud, T. Gudbjartsson

**Affiliations:** 1 Department of Vascular Surgery, Haukeland University Hospital, Jonas Lies vei 65, P.O. Box 1400, 5021 Bergen, Norway; 2 Western Norway Trauma Centre, Haukeland University Hospital, Bergen, Norway; 3 Department of Cardiothoracic Surgery, Landspitali University Hospital, Reykjavík, Iceland; 4 Faculty of Medicine, University of Iceland, Reykjavík, Iceland

**Keywords:** Vascular trauma, Injury score, Blunt, Penetrating, Scandinavian, Mortality, Treatment

## Abstract

**Background:**

Outcomes after vascular injuries in wartime are well documented, but studies on vascular injuries in a civilian European populations are scarce.

**Methods:**

A retrospective study on all adults admitted to a North-European level 1-trauma centre 2009–2018 with The Abbreviated Injury Scale-codes for non-iatrogenic vascular trauma (VT). Data were extracted from both national and regional trauma-registries, as well as patient charts. Patient demographics, mechanism, and location of vascular injury were registered as well as its treatment. Incidence and injury scores (ISS, NISS and TRISS) were calculated and overall survival (Kaplan–Meier) estimated.

**Results:**

Of 4042 trauma-patients, 68 (1.7%) (median age 44 years, 76% males) sustained 81 vascular injuries (69 arterial; 12 venous); 46 blunt and 22 (32%) penetrating injuries. The total incidence of vascular injuries was 1.45/100,000 inhabitants and did not change over the study-period (95% confidence interval 1.13–1.82). The injuries were located in thorax (n = 17), neck (n = 16) and abdominal region (n = 15); most of the blunt injuries followed traffic (n = 31) or falling accidents (n = 10), and with 17 of the 22 penetrating injuries due to stabbing. The median ISS and NISS-scores were 22 and 33, with 50 (74%) and 55 (81%) patients having scores > 15, respectively. Forty-three (63%) patients had open surgical repair and 8 (12%) received endovascular treatment. Twenty-one patients died within 30-days (31%), 33% and 27% after blunt and penetrating injuries, respectively. Half of the patients that died within 24 h sustained aortic injury.

**Conclusions:**

Traumatic vascular injuries are rare in civilian settings and are less than 2% of major trauma admissions. These patients are often seriously injured and their treatment can be challenging with high 30-day mortality.

**Trial registration:** Retrospectively registered.

## Introduction

Trauma due to violence and injury kills nearly half a million people in Europe every year and many more are injured [1]. Even though progress has been made in prehospital care, injury prevention, automotive safety and emergency medical services, the proportion of deaths occurring immediately after trauma still remains at 50–60% [2]. Injuries still account for 5.3% of all deaths in Europe and are a leading cause of death in people aged 15–29 years, according to the World Health Organization latest report [1]. In Norway approximately 6% of all deaths each year are injury-related [3]. Vascular injuries are common in severe trauma and may be responsible for up to 20% of trauma deaths [4, 5]. Vascular trauma (VT) has been well documented during wartime but epidemiological studies in civilian setting are scarce, both for blunt and penetrating vascular injuries [6–8]. Still the incidence of vascular trauma has been reported between 1.6 and 2% of adults admitted for major trauma during peacetime [9].

Trauma involving the major arteries and veins is a major cause of morbidity and mortality. The highest mortality is due to transection of the thoracic aorta and severe injuries to the abdominal vessels [10, 11]. The patients who reach hospital alive place a significant burden on hospital resources and have the highest utilization among trauma patients of blood transfusion, critical care and hospital stay; especially following blunt trauma [4, 5, 12].

The conventional treatment of vascular trauma has been open surgical repair, with or without the use of vascular prosthesis [10]. Minimally invasive endovascular techniques, such as stent graft insertion and embolization with coils, glue or gel foam, are now more frequently applied in the treatment of vascular injury in acute trauma [13]**,** however the outcomes of these treatments are not well recorded in peacetime in Europe [14].

The aim of this retrospective study was to document the incidence, treatment and early (30 day) outcome of admitted non-iatrogenic adult civilian vascular trauma patients in in a level-1 North European trauma centre serving a well-defined geographical region.

## Material and methods

### Study design

This was a retrospective analysis of all patients ≥ 18 years of age sustaining vascular trauma and admitted alive on admission and registered in the trauma registry at Haukeland University Hospital in Bergen between 1st January 2009 and 31st December 2018. This research was registered and approved by the regional committees for medical and health research ethics in Norway under file number 2017/293.

### Trauma care in Norway

Haukeland University Hospital functions both as a local hospital for the city of Bergen and surrounding areas and is the regional trauma centre in Western Norway. Patients admitted to other hospitals in the region and subsequently transferred to the regional trauma centre fulfilling the above inclusion criteria were also included in the study. We assume that most severe vascular injuries are transported directly to our institution, or transferred from local hospitals following initial trauma evaluation and resuscitation.

When a patient is classified as a trauma patient and admitted to our hospital the trauma team evaluates and treats the patient according to the American College of Surgeons Advanced Trauma Life Support courses (ATLS^®^) guidelines [15]. The purpose of the team is to provide advanced simultaneous care from relevant specialists to the seriously injured patient. The trauma team comprises a multidisciplinary group of individuals drawn from the specialties of surgery, anaesthesia and support staff [16]. The trauma-team leader is usually an experienced surgical resident, who if needed consults the senior in-house trauma surgeon on call. Based on the spectre of injuries, other consultants as vascular, orthopaedic, cardiothoracic, neuro- or gastrointestinal surgeons may be involved.

### Study population and data sources

Data from 1st January 2009 to 31st December 2015 with the Abbreviated Injury Scale (AIS) for non-iatrogenic vascular trauma were collected from The Local Trauma Registry at Haukeland University Hospital and data from 1st January 2016 to 31st December 2018 from The National Trauma Registry. Baseline demographic information and clinical data were gathered from pre-hospital reports, patient charts, surgical descriptions, and autopsy reports using a standardized data sheet. Inclusion criteria were presence of vascular trauma on named artery or vein. Excluded were iatrogenic injures and isolated vascular injures to the head and solid organs in the abdomen. Patients pronounced dead before and on arrival to hospital were excluded. Mechanism and location of injury, surgical treatment, along with the length of hospital stay were registered.

### Classification of vascular injuries and injury score

Polytrauma was defined as having The Abbreviated Injury Scale of ≥ 3 in two or more body regions. Hypovolemic shock was defined as a systolic blood pressure (SBP) < 90 mmHg or base deficit > 6, or a blood transfusion requirement > 4 units within the first 24 h. Massive transfusion was defined as a transfusion requirement of ≥ 10 units packed red blood cells within 24 h. Primary repair included patch angioplasty, direct suture repair or end-to-end anastomosis.

Based on physiological status from injury at admission, the severity score (ISS and NISS) and revised trauma score (RTS) were calculated and used to estimate the probability of survival from the Trauma Score–Injury Severity Scores (TRISS). In the predetermined equation for TRISS the age of the patient and whether the injury was blunt or penetrating, are taken into account [17].

### Endpoints

The primary outcome was 30 day and long-term mortality. All the trauma registry data were followed-up as long as one year or until death, follow-up being 96% complete. Three patients were transported or moved out of Norway and therefore complete one year follow-up was impossible.

Secondary outcome measures and hospital resource use were measured in terms of hospital length of stay (LOS, days), intensive care unit (ICU) length of stay and blood transfusion requirements in the first 24 h.

### Statistical analyses

Microsoft Excel (Office 16) and RStudio (4.0.2) were used for descriptive statistics. Categorical data were presented as counts and percentages, continuous data as median and IQR. Fisher-Exact or the Chi-squared test were used where applicable, and independent *t*-test or Mann Whitney U test applied as appropriate. A value of *p* < 0.05 was considered statistically significant. Incidence was calculated according to the age and gender distribution of the population as derived from National Statistics Norway, standardized to the WHO Standard European Standard Population and the annual percent change in incidence by residency estimated using the Poisson regression. Odds ratios (ORs) for predictors of short-term mortality (< 24 h or 30-day mortality) were estimated with logistic regression and difference in survival by gender estimate by log-rank test. Overall survival was estimated with the Kaplan–Meier method and difference in survival by gender estimate by log-rank test.

## Results

### Patient demographics

A total of 4042 patients were admitted as trauma patients to our level 1 centre and evaluated by our trauma team; including 68 patients that met criteria for vascular trauma with a total of 81 vascular injuries; 69 arterial and 12 venous. Baseline patient characteristics are shown in Table 1. There were 52 males and 16 females (M/F ratio: 3.25), the median (IQR) age for all patients being 44 (31–60) years, and 42 (31–60) and 50 (40–63) years, for males and females, respectively.

**Table 1:** Demographical and clinical characteristics of vascular patients by mechanism of injury

	Vascular trauma			
	Total	Penetrating	Blunt	*p* value
*Demographics*				
Number of patients (%)	68	22 (32)	46 (68)	< 0.05
Age (mean, SD)	44 (17)	41 (16)	48 (18)	0.14
Male (%)	52 (76)	19 (86)	33 (72)	0.23
LOS days* (median, IQR)	4 (1.5–15.5)	3.5 (1–6)	10 (3–21)	0.08
ICU days** (median, IQR)	3.5 (2–9)	1.5 (1–3)	4.5 (3–10)	0.12
ICU number of patients (%)	34 (50)	8 (36)	26 (57)	0.19
*Injury characteristics*				
ISS (median, IQR)	22 (14–36)	14 (8–17)	32 (20–38)	< 0.05
NISS (median, IQR)	33 (17–46)	17 (10–27)	34 (27–48)	< 0.05
ISS > 15 (%)	50 (74)	10 (45)	40 (87)	< 0.05
Vascular AIS ≥ 3 (%)	47 (69)	13 (59)	34 (74)	0.27
Polytrauma (%)	32 (47)	1 (5)	31 (67)	< 0.05
Hypovolemic shock (%)	34 (50)	13 (59)	21 (46)	0.44
Massive transfusion of PRBC*** (%)	12 (18)	4 (18)	8 (17)	1.00

Data are presented as Mean (SD) or Median (IQR) or frequency (%)

*LOS length of stay

**ICU days for those admitted to the ICU

***Packed red blood cells

### Incidence

**Fig. 1:**
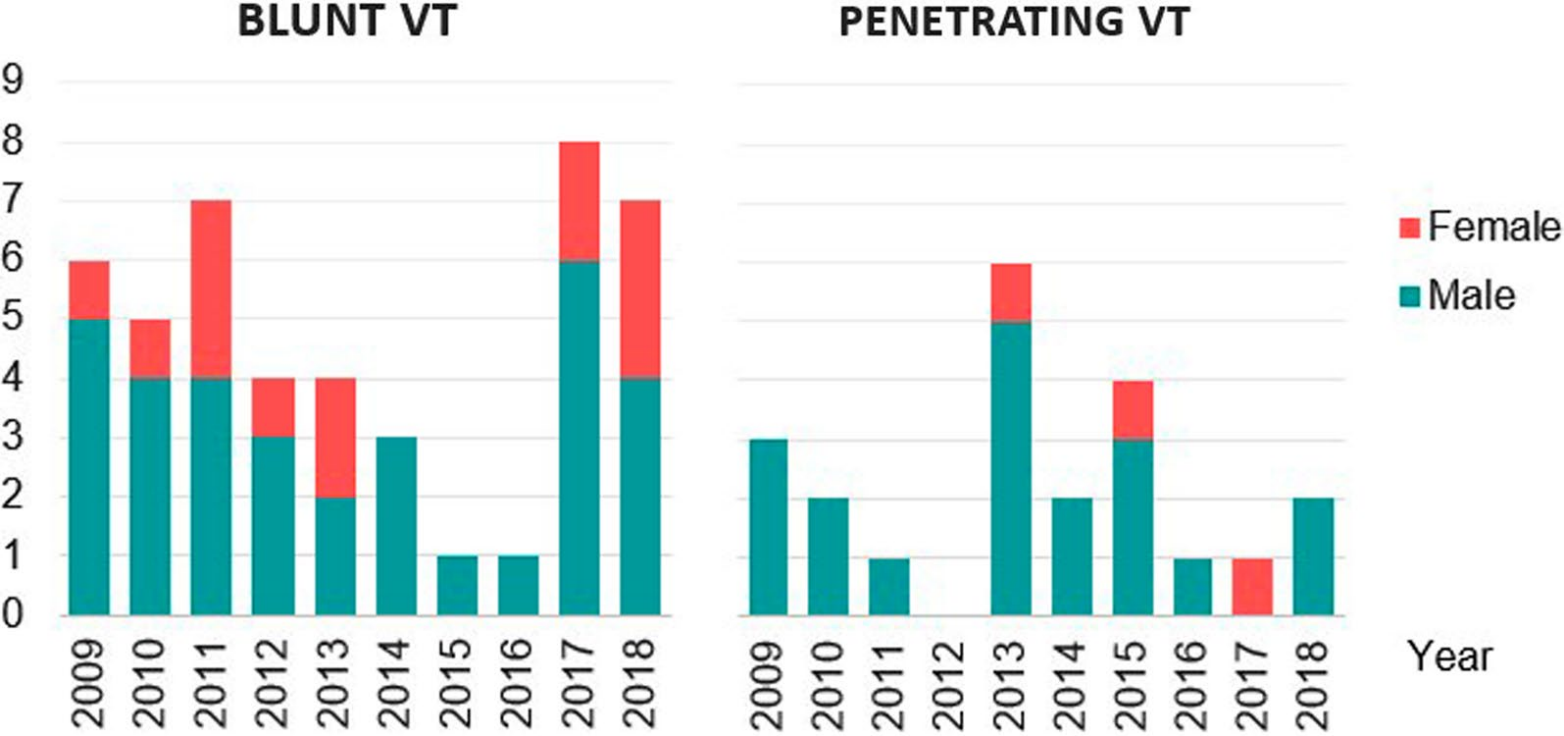
Distribution of the admitted patients over the study period with blunt and penetrating vascular trauma (VT)

**Fig. 2:**
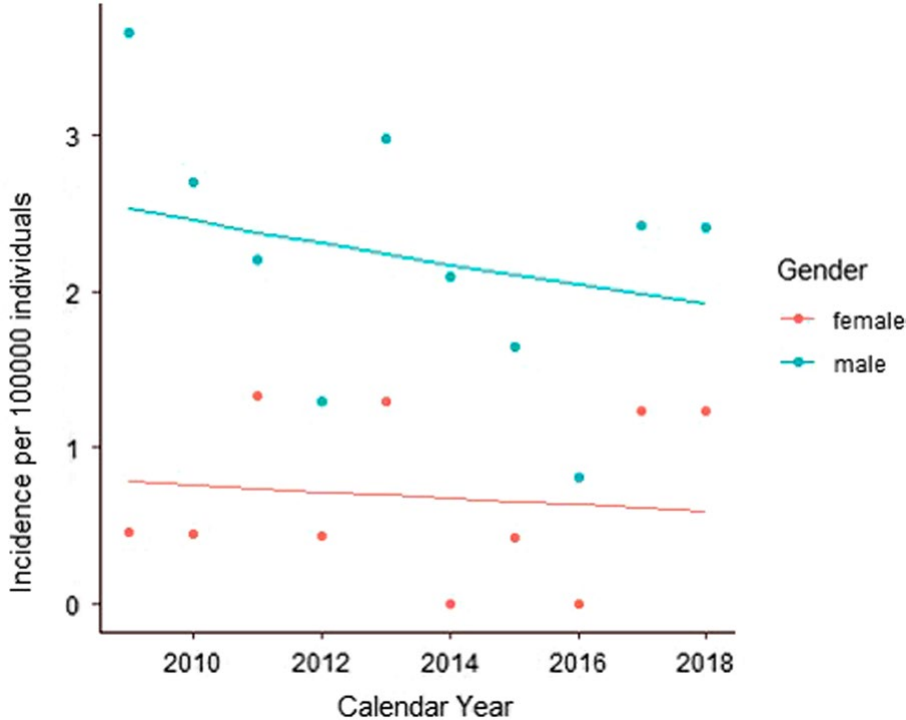
Incidence of vascular trauma admitted to hospital evaluated and treated by the trauma team in males (blue) and females (red) 2009–2018. The total incidence was 1.45 per 100,000 inhabitants (95% confidence interval: 1.13–1.82) with no significant rate change over the 10-year study period (p = 0.48)

The number of patients admitted annually with vascular injuries is shown in Fig. 1. The total incidence was 1.45 per 100,000 inhabitants (95% confidence interval 1.13–1.82) with annual incidence of 0.97 per 100,000 inhabitants (95% confidence interval 0.893–1.054) with no significant rate change over the 10-year study period (*p* = 0.48). Furthermore, there was no significant difference in 30 day (*p* = 0.52) or one year survival between males and females (*p* = 0.57) (Fig. 2).

### Location of injuries and vessels involved

**Fig. 3:**
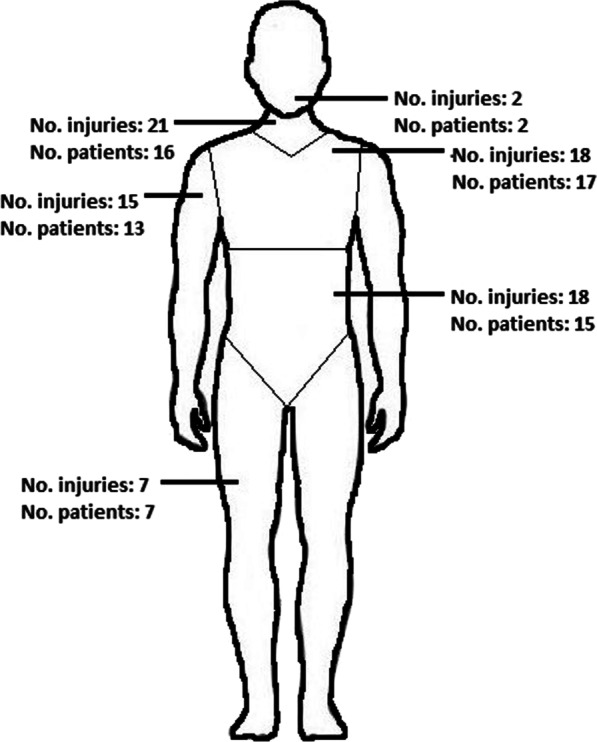
The location of vascular trauma that included 81 vascular injuries in 68 patients

Most patients had vascular injury located to the chest (25%; n = 17) or to the neck (24%, n = 16), followed in frequency by injuries to the abdominal region (22%, n = 15). (Fig. 3.)

Out of the 81 vascular injuries, 69 involved arteries and 12 veins, including 8 patients (12%) with isolated venous injury and three with combined arterial and venous injury. Table 2 shows the most common vessels injured that was the thoracic (n = 11) followed by the abdominal aorta (n = 4).

**Table 2:** Anatomical location of vascular injury and how they were treated

AIS anatomical zone	Name of artery/vein	Number of patients*	Number of injuries (n = 81)			Open operation			Endovascular treatment	
			Total	Penetrating	Blunt	Ligation	Primary repair	Interposition graft	Embolisation	Stent/stentgraft
Face	*A. carotis externa*	2	2	1	1	2	–	–	–	–
Neck	*A. carotis externa*	1	1	1	0	1	–	–	–	–
	*A. carotis communis/interna*	10	11	1	10	–	1	–	–	–
	*A. vertebralis*	5	6	0	6	–	–	–	–	–
	*V. jugularis interna*	1	1	1	0	–	–	–	–	–
	*V. jugularis ext*	2	2	2	0	1	–	–	–	–
Thorax	*Thoracic aorta*	11	11	1	10	–	–	1	–	5
	*A. brachiocephalic*	1	1	1	0	–	1	–	–	–
	*A. bronchial/esiophageal/intercostal/internal mammary*	3	3	1	2	1	1	–	–	–
	*V. cava superior*	1	1	0	1	–	–	–	–	–
	*V. azygos/bronchial/esophageal/ hemaizygos/intercostal/internal jugular/internal mammary*	2	2	0	2	–	–	–	–	–
Abdomen	*Abdominal aorta*	4	4	1	3	–	–	–	–	–
	*A. hepatic/renal/splenic*	5	5	2	3	–	–	–	–	–
	*A. mesenterica superior*	2	2	2	0	1	1	–	–	–
	A. iliac	4	4	0	4	–	–	–	3	
	*V. cava inferior*	1	1	0	1	–	1	–	–	–
	*V. portal/renal/splenic/superior mesenteric*	2	2	1	1	–	1	–	–	–
Upper extremity	*A. brachialis*	4	4	2	2	–	3	1	–	–
	*A. radialis/ulnaris*	8	10	9	1	2	5	1	–	–
	*V. axillaris*	1	1	1	0	1	–	–	–	–
Lower extremity	*V. femoralis*	1	1	0	1	1	–	–	–	–
	*A. tibialis/fibularis*	2	2	0	2	1	–	–	–	–
	*A. poplitea*	3	3	0	3	–	1	2	–	–
	*V. saphena magna*	1	1	1	0	1	–	–	–	–

Patient can have more than one vascular injury

### Mechanism of injury

Blunt injury was significantly more common than penetrating (68% vs. 32%, *p* < 0.05). Most blunt injuries resulted from traffic accidents (n = 31) that included 20 patients as a driver/passenger in a car, 8 moped/motorcycle/bicycle accidents and 3 pedestrians. Ten patients were involved in a fall accident from a height over 3 m. Out of 22 penetrating injuries, 17 were stabbing injuries, all with a knife and none involving fire arm. (Table 3).

**Table 3:** Mechanism of injury for the 68 vascular trauma patients. Pedestrian relates individual who are walking and involved in road traffic accident

Mechanism of injury	No. patients	%
Car	20	29
Knife	17	25
Fall from height > 3 m	10	15
Motorcycle/moped	7	10
Penetrating (other)	5	7
Pedestrian	3	4
Crushing	3	4
Strangulation	2	3
Cyclist	1	1

Penetrating trauma (other) was injury due to glass, electric saw, axe, and metal objects in an explosion

### Severity of injury

The median ISS and NISS values of the 68 patients who were admitted were 22 (14–36) and 33 (17–46), respectively; with 50 (74%) and 55 (81%) of the patients with ISS and NISS scores above 15; indicating severe injury. ISS and NISS scores were significant higher for blunt trauma, or 32 (20–38) compared to 34 (27–48) for penetrating trauma 14 (8–17) and 17 (10–27) (*p* < 0.05). The median estimated TRISS was 91%, or 93% vs. 90% (*p* = 0.90) for penetrating vs. blunt trauma; respectively.

Only one patient with penetrating injury suffered polytrauma (AIS of ≥ 3 in two or more body regions) compared to 67% (n = 31) of the blunt trauma patients (*p* = 0.27) (Table 1).

### Hospital resources

Hypovolemic shock was registered for 50% of the patients; similar in penetrating (59%) and blunt vascular trauma (46%) (*p* = 0.44). During the trauma evaluation at our institution, 60% of the patients underwent trauma CT imaging, and in 25% of cases patients were taken directly to the operating room for emergency surgery. Eight patients were transported to our level 1 trauma centre from another hospital, and in all those cases a trauma CT imaging had been completed.

Blood transfusions were administered to 45 patients (17/22 of the penetrating and 28/46 of the blunt trauma cases); transfused patients received a median of 5 units (IQR 2.5–10.5, range 1–82); 6.5 (IQR 3–10.5) and 4 (IQR 2–9) units for blunt and penetrating trauma, respectively (*p* = 0.90).

Massive PRBC transfusion (≥ 10 units PRBCs within 24 h.) was required for 18% of the patients (12/68) (Table 1). The median hospital stay for all admitted patients was 4.5 days (2–16); but there was no significant difference between patients admitted with penetrating trauma and blunt trauma (3.5 (1–6) vs.10 days (3–21) (p = 0.08)). Furthermore, there was no significant difference in the length of ICU stay between the penetrating and blunt groups (1.5 (1–3) vs. 4.5 (3–10) (*p* = 0.12)) (Table 1)

### Treatment

Forty three of the 68 patients (63%) were treated surgically; 86% (n = 19) of the patients with penetrating trauma, and 52% (n = 24) of patients with blunt injury. The different procedures for each injury, both blunt and penetrating, are shown in Table 2. Emergency thoracotomy was performed on 9 (13%) of the patients; five with penetrating and four with blunt trauma. Emergency thoracotomy of two of the penetrating trauma were performed in the emergency room, the other seven in an operating room close to the emergency room. Primary repair (n = 15) was the most common procedure; 12 injured vessels being ligated and 5 operated on with an interposition of a graft. No patient needed to be amputated as a result of the vascular injury.

**Fig. 4:**
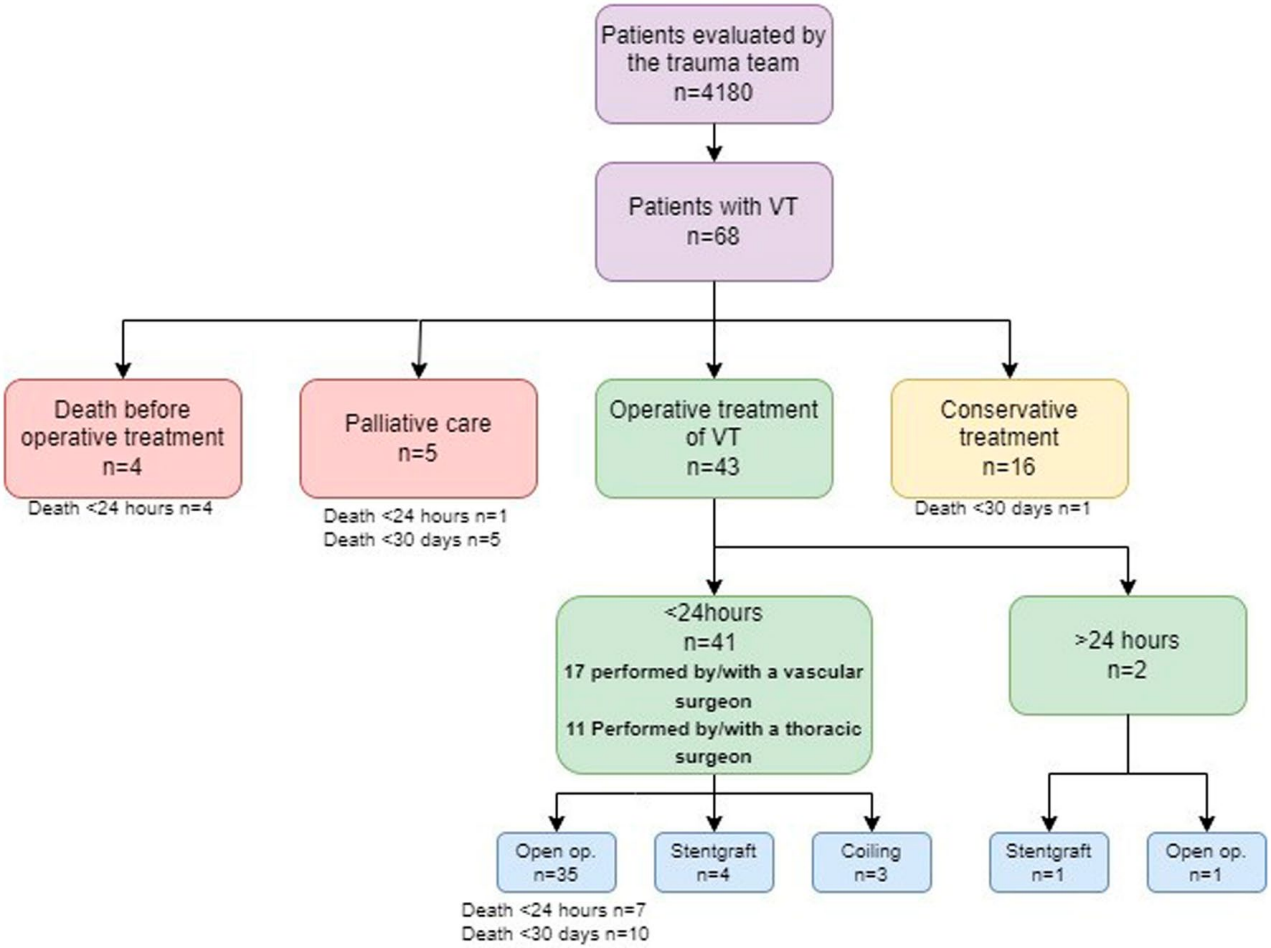
Flow chart showing the management of, and outcome in 68 patients with vascular trauma (VT) in western Norway 2009–2018

In 8 (12%) of the patients; all of them with a blunt injury, the bleeding could be stopped with an endovascular repair; the procedure being performed in all cases except one within 24 h. This included stenting in five cases and coiling in three (Fig. 4).

Four patients died before surgical / endovascular treatment and 16 received only conservative treatment; including most of the injuries to the common/internal carotid or vertebral arteries. Of the four abdominal aortic injuries; two had a traumatic aortic dissection and were treated conservatively, but two patients died during an emergency open procedure. Palliation was chosen for five of the patients, most often due to serious brain injury (Fig. 4).

A majority of the operations were performed by a vascular surgeon (n = 17); including two cases together with an endovascular radiologist, but in 6 cases with a cardiothoracic surgeon and in 6 other cases with surgeons from other surgical disciplines. Vascular reconstruction was performed solely by a general surgeon in 6 cases.

### Short and long-term mortality

**Fig. 5:**
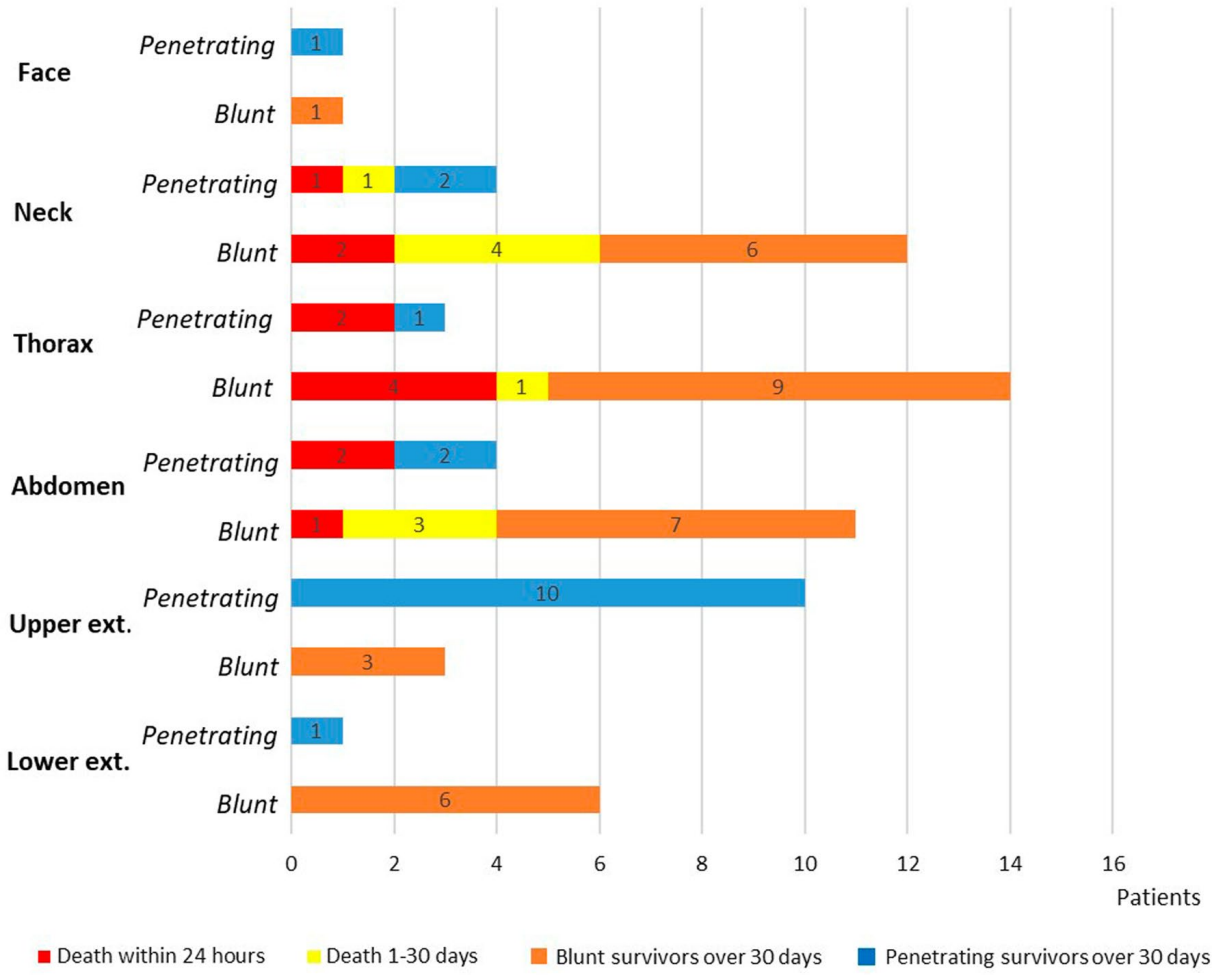
The location of the vascular trauma for patients with penetrating (blue column) and blunt injury (orange column); together with mortality within 24 h (red column) and within 30 days (red and yellow column)

There was no significant difference in 24 h. (18%; 23% vs. 15%, log-rank test, *p* = 0.45) or 30 day mortality (31%; 27% vs. 33%, log-rank test, *p* = 0.71) between penetrating and blunt injury. All but one of the 6 patients who died due to penetrating injury succumbed within 24 h after admission (Fig. 5). There was no significant difference in 30 day mortality between males and females (29% vs. 38%, log-rank test, *p* = 0.50).

Of the patients who underwent emergency thoracotomy, 78% (7/9) died; all of them within 24 h. The mortality rate related to aortic injury was 40% (thoracic (4/11); abdominal (2/4)) and all those who died due to aortic trauma died within 24 h of admission. Furthermore, both patients with penetrating aortic injuries died.

### Cause of death

Of the 22 patients who died within one year, an autopsy was performed in 17 (77%) of the cases. Vascular trauma was the major cause of death in 15 of these patients; including 12 that died due to haemorrhagic shock and three because of ischemic injury to the brain. Haemorrhagic shock was the cause of death in all except one (11/12) of the patients who died within 12 h. Vascular trauma was unrelated to death in six patients (1 died within 24 h and 5 within 30 days), but one of these patients did not have a registered cause of death 7 months after trauma admission.

### Late survival

The long-term survival of the 68 individuals with vascular trauma was 67% at 1 year. There was no significant difference between males and females (69% vs. 62%, log-rank test, *p* = 0.57), or between penetrating and blunt trauma (72% vs. 65%, log-rank test, *p* = 0.6).

## Discussion

This study is the first to characterize the epidemiology of vascular trauma within an organized Scandinavian trauma system. Traumatic vascular injury was present in 1.7% of patients admitted for serious trauma with a significantly higher proportion following blunt compared to penetrating trauma. Both the 24-h and 30-day mortality were high, 18% and 30%, respectively, underscoring the severity of these injuries. Around 80% of the patients who were admitted and evaluated at arrival of the HUS trauma team, had ISS/NISS scores above 15; a threshold number that reflect severe and life threating injuries. Open surgery was the main form of treatment, especially for cases of penetrating vascular trauma, where primary repair without graft was the most used technique, and endovascular techniques and in some cases conservative management were reserved for selected cases.

Reports from trauma centres in Australia and the United States (US) describe vascular trauma as responsible for between 1% and 2.5% of general trauma admissions [4, 5, 18]. The incidence of vascular injury in the current study (1.7%) was similar to reports from other countries with organized trauma systems. Even though some cases may have gone unreported in the current study, our incidence figures of vascular trauma provide a realistic assessment of the true incidence in Western Norway.

In contrast to the United States, and some other countries where incidence of penetrating trauma is high, a most vascular injuries in Northern Europe is caused by blunt trauma. As the actual incidence of vascular trauma in most European countries is still unknown, [14] hopefully this study might shed some more light upon this issue. Our incidence figures are in line with those from Canada and Australia where 63–68% of vascular trauma is due to blunt force trauma [19, 20], whereas in Finland, Sweden, United States and United Kingdom blunt trauma is reported to be the cause of vascular trauma in 38–48% of the cases [21–23]. Blunt trauma following a motor vehicle accident represented 46% of our vascular trauma cases. Actually vascular trauma is subject to geographic differences and highly depends on specific trauma mechanism where traffic accidents are predominantly associated with blunt injuries [24, 25]. Compared to the US, where firearm injury is a leading cause of trauma related deaths, [26, 27], these are rare in Norway, and interestingly no penetrating injuries in our patient cohort were due to shooting.

Injury scores in our study were significant higher for blunt vs. penetrating injury; but 67% of the blunt vascular trauma suffered polytrauma and often were in hypovolemic shock when admitted; a finding that is in line with other similar studies [5, 20]. Still, no significant differences were identified for LOS or need for blood transfusion in the two groups groups; possibly due to insufficient power (type II error). A vascular trauma following blunt trauma as compared to penetrating injury, is a marker of significant transmission of force and therefore often substantially more severe injury with more extensive damage to associated soft tissue, bones, and nerves [5, 20, 28, 29].

The current study highlights the high morbidity and mortality associated with vascular trauma. In-hospital mortality was 29%, which is higher than observed mortality in other major trauma centres that have reported figures in the 5–23% range [5, 20, 22, 30]. This difference between these studies could to some extent be explained by the fact that in our hospital patients with less severe vascular trauma are not defined as polytrauma patients and thus not evaluated by the trauma team—and therefore not included in the present study.

Of those patients who died, vascular trauma was the reason for death in 68% of the patients, this according to the autopsy reports. Exsanguination is the most significant cause of potentially preventable death after injury [31], and in our study haemorrhagic shock was the dominant cause of death in 12 of 22 (55%) patients. The one-year mortality in this series being 32%, but interestingly, none of the patients suffering from extremity vascular trauma died during the study period. .

When evaluating polytrauma outcome, the TRISS is the most applied system. It combines injury assessments based on physiological (RTS) and anatomical (ISS) status, with age and trauma mechanism (blunt or penetrating) to calculate the safety probability [32, 33]. However, according to a study conducted by Shang et al., RTS and TRISS scoring systems do not weight factors specific for vascular injuries, thus underestimate mortality in the polytrauma patient with vascular injury [34].

The most common vascular trauma involved an injury to the aorta (22% of the cases), most of them to the thoracic aorta (75%) and usually following a blunt injury (87%). These injuries are serious, as reflected in the fact that the patients who died from aortic injury (40% of the cases), died within 24 h of arrival in the emergency department. This is in line with other similar studies, including the one by Jamieson et al. who reported that 50% patients who live to be evaluated in a hospital suffering thoracic aortic injury die within the 24 h [35].

Although. 60% (9/15) of patients with aortic injuries were operated on within the first 24 h and four of those were treated with an endovascular stent graft. All of the patients treated with endovascular stent graft survived 30 days, as well as the first year after sustaining vascular trauma. Recent studies have indicated that treatment with thoracic endovascular aortic repair (TEVAR) have decreased mortality, especially due to blunt thoracic aortic injury [36–38]. However, it should still be stressed that although endovascular treatment in vascular trauma has both gained acceptance and its usage has increased in the past decade, some international Society guidelines only recommend it in blunt thoracic injury; see: Society for Vascular Surgery (SVS) (Grade 2, Level C) and European Society for Vascular Surgery (ESVS) (Class 1, Level C) [37, 39, 40]. Endovascular treatment may offer greater versatility and the ability to treat anatomic areas difficult to reach with open surgery [41]. The procedure must be performed in specially equipped operating theatres with highly specialized staff that is not available in all hospitals, not even all tertiary trauma care centres. Currently, there is a shortage of endovascular physicians involved in trauma in some regions [42]. At Haukeland University Hospital, however, an experienced radiologist is always on call, and a hybrid suite available for acute cases. In our study all procedures were performed with an interventional radiologist and a surgeon together.

The majority of vascular trauma patients were still treated surgically, most often by vascular (n = 17) or thoracic surgeons (n = 11). In the current cohort, the most common surgical procedure was simple suturing or ligation, which is in line with earlier reports [21]. Importantly, none of the patients suffering from extremity vascular trauma needed an amputation during the study period.

## Strength and limitations

The main strength of this study was the study cohort from a well-defined area in Norway. A further strength was the fact that we could access both a national and local trauma registry with near complete follow-up.

Being a retrospective study and therefore potentially subject to selection bias is a limitation, though from a prospectively maintained database, is a limitation. Also, our cohort represents a relatively small number of cases and only covered vascular trauma in adult patients. Consequently, the age-standardized incidence does not represent all ages. Furthermore, only hospitalized patients were analysed, and not those who died on the scene or during transport to a hospital.

## Conclusion

Severe vascular trauma is relatively uncommon (1.7%) in civil settings in Western Norway; but still carries a significant mortality with an 18% and 30% 24-h and 30 day mortality, respectively. Major vascular trauma is most often caused by motor vehicle accidents in this cohort, falls, knife stabbings and remarkably not gunshot injuries. Most of the vascular injury was due to blunt trauma unlike in USA and UK. However, our treatment outcome is similar to internationals reports from high volume level 1-trauma centres. Vascular trauma injuries are often challenging, especially regarding diagnostics and therapy and are associated with high morbidity and mortality, and burden on hospital resources. Despite advances in endovascular technology and availability, the majority of vascular trauma are still treated with open surgery.

## Data Availability

The data that support the findings of this study are available from Haukeland University Hospital, and the Western Norway Trauma Centre, but restrictions apply to the availability of these data, which were used under license for the current study, and so are not publicly available. Data are however available from the authors upon reasonable request and with permission of The regional committees for medical and health research ethics in Norway and Haukeland University Hospital, and the Western Norway Trauma Centre.

## Abbreviations

AIS: Abbreviated injury scale
ICU: Intensive care unit
ISS: Injury severity score
IQR: Interquartile range
LOS: Length of stay
NISS: New injury severity score
SBP: Systolic blood pressure
PRBCs: Packed red blood cells
RTS: Revised traumas score
TEVAR: Thoracic endovascular aortic repair
TRISS: Trauma score–injury severity scores
VT: Vascular trauma

## References

[1] Yon Y, Hernandez-Garcia L, Di Giacomo G (2020). Reducing violence and injury in the WHO European region. Lancet Public Health.

[2] Sobrino J, Shafi S (2013). Timing and causes of death after injuries. Proceedings.

[3] Health NIoP. Public Health Report fhi.no: NIPH; 2017 [updated 18.12.2017]. Available from: https://www.fhi.no/en/op/hin/injuries/injuries-in-Norway/#:~:text=About%202%20500%20people%20die,remainder%20are%20mainly%20from%20suicide.

[4] Sugrue M, Caldwell EM, Damours SK (2002). Vascular injury in Australia. Surg Clin North Am.

[5] Perkins ZB, De'Ath HD, Aylwin C (2012). Epidemiology and outcome of vascular trauma at a British major trauma centre. Eur J Vasc Endovasc Surg.

[6] Humphrey PW, Nichols WK, Silver D (1994). Rural vascular trauma: a twenty-year review. Ann Vasc Surg.

[7] Oller DW, Rutledge R, Clancy T (1992). Vascular injuries in a rural state: a review of 978 patients from a state trauma registry. J Trauma.

[8] Mattox KLFD, Burch J (1989). Five thousand seven hundred and sixty cardiovascular injuries in 4459 patients: epidemiological evolution 1958 to 1987. Ann Surg.

[9] Szaniewski KBT, Sikora T. Vascular trauma London: IntechOpen. 2019. [Available from: https://www.intechopen.com/chapters/68195.

[10] Callcut RA, Mell MW (2013). Modern advances in vascular trauma. Surg Clin North Am.

[11] Bui TD, Mills JL (2009). Control of inferior vena cava injury using percutaneous balloon catheter occlusion. Vasc Endovascular Surg.

[12] Caps MT (1998). The epidemiology of vascular trauma. Semin Vasc Surg.

[13] Coimbra LKAR. Vascular trauma: new directions in screening, diagnosis and management. In: Yamanouchi DD, editor. Vascular surgery. 2012.

[14] Fingerhut A, Leppaniemi AK, Androulakis GA (2002). The European experience with vascular injuries. Surg Clin North Am.

[15] Subcommittee A, American College of Surgeons' Committee on T, International Awg. Advanced trauma life support (ATLS(R)): the ninth edition. J Trauma Acute Care Surg. 2013;74(5):1363–6. 10.1097/TA.0b013e31828b82f5 23609291

[16] Georgiou A, Lockey DJ (2010). The performance and assessment of hospital trauma teams. Scand J Trauma Resusc Emerg Med.

[17] Boyd CR, Tolson MA, Copes WS (1987). Evaluating trauma care: the TRISS method. Trauma score and the injury severity score. J Trauma.

[18] Gupta R, Rao S, Sieunarine K (2001). An epidemiological view of vascular trauma in Western Australia: a 5-year study. ANZ J Surg.

[19] Smith S, Allen L, Khwaja K (2022). Management of vascular trauma across Canada: a cohort study with implications for practice. Injury.

[20] Weller J, Bowles M, Summers Z (2021). The epidemiology and outcomes of vascular trauma in Gold Coast, Australia: institutional experience at a level 1 trauma centre. ANZ J Surg.

[21] Poyhonen R, Suominen V, Uurto I (2015). Non-iatrogenic civilian vascular trauma in a well-defined geographical region in Finland. Eur J Trauma Emerg Surg.

[22] DuBose JJ, Savage SA, Fabian TC (2015). The American association for the surgery of trauma prospective observational vascular injury treatment (PROOVIT) registry: multicenter data on modern vascular injury diagnosis, management, and outcomes. J Trauma Acute Care Surg.

[23] Barmparas G, Inaba K, Talving P (2010). Pediatric vs adult vascular trauma: a national trauma databank review. J Pediatr Surg.

[24] Wani ML, Ahangar AG, Wani SN (2012). Peripheral vascular injuries due to blunt trauma (road traffic accident): management and outcome. Int J Surg.

[25] Meyer A, Huebner V, Lang W (2020). In-hospital outcomes of patients with non-iatrogenic civilian vascular trauma. Vasa.

[26] Department SR. Number of people killed by firearms in Norway 2012–2020 statista.com: Statista Research Department; 2022 [Available from: https://www.statista.com/statistics/670978/number-of-people-killed-by-firearms/.

[27] Klein J, Prabhakaran K, Latifi R (2022). Firearms: the leading cause of years of potential life lost. Trauma Surg Acute Care Open.

[28] Rozycki GS, Tremblay LN, Feliciano DV (2003). Blunt vascular trauma in the extremity: diagnosis, management, and outcome. J Trauma.

[29] Tan TW, Joglar FL, Hamburg NM (2011). Limb outcome and mortality in lower and upper extremity arterial injury: a comparison using the National Trauma Data Bank. Vasc Endovascular Surg.

[30] Altoijry A, Lindsay TF, Johnston KW (2021). Vascular injury-related in-hospital mortality in Ontario between 1991 and 2009. J Int Med Res.

[31] Kauvar DS, Wade CE (2005). The epidemiology and modern management of traumatic hemorrhage: US and international perspectives. Crit Care.

[32] Hadisaputra IHSG, Mahadewa TG (2021). Adjustment of trauma and injury severity sccore (TRISS) and revised trauma score (RTS) in predicting mortality of multitraumapatients in Sanglah hospital bali. Biomed Pharmacol J.

[33] Domingues CA, Coimbra R, Poggetti RS (2018). New trauma and injury severity score (TRISS) adjustments for survival prediction. World J Emerg Surg.

[34] Loh SA, Rockman CB, Chung C (2011). Existing trauma and critical care scoring systems underestimate mortality among vascular trauma patients. J Vasc Surg.

[35] Jamieson WR, Janusz MT, Gudas VM (2002). Traumatic rupture of the thoracic aorta: third decade of experience. Am J Surg.

[36] Shalhub S, Starnes BW, Brenner ML (2014). Blunt abdominal aortic injury: a Western Trauma Association multicenter study. J Trauma Acute Care Surg.

[37] Reuben BC, Whitten MG, Sarfati M (2007). Increasing use of endovascular therapy in acute arterial injuries: analysis of the National Trauma Data Bank. J Vasc Surg.

[38] Branco BC, DuBose JJ, Zhan LX (2014). Trends and outcomes of endovascular therapy in the management of civilian vascular injuries. J Vasc Surg.

[39] Riambau V, Bockler D, Brunkwall J (2017). Editor's choice—management of descending thoracic aorta diseases: clinical practice guidelines of the European society for vascular surgery (ESVS). Eur J Vasc Endovasc Surg.

[40] Lee WA, Matsumura JS, Mitchell RS (2011). Endovascular repair of traumatic thoracic aortic injury: clinical practice guidelines of the society for vascular surgery. J Vasc Surg.

[41] Glaser JD, Kalapatapu VR (2019). Endovascular therapy of vascular trauma-current options and review of the literature. Vasc Endovascular Surg.

[42] Matsumura Y, Taudorf M, Søvik E, Hörer T, DuBose JJ, Rasmussen TE, White JM (2020). Endovascular resuscitation and trauma management: education and simulation. Endovascular resuscitation and trauma management: bleeding and haemodynamic control.

